# A Placebo-Controlled Trial of Dextromethorphan as an Adjunct in Opioid-Dependent Patients Undergoing Methadone Maintenance Treatment

**DOI:** 10.1093/ijnp/pyv008

**Published:** 2015-03-06

**Authors:** Sheng-Yu Lee, Shiou-Lan Chen, Yun-Hsuan Chang, Chun-Hsien Chu, Shih-Heng Chen, Po See Chen, San-Yuan Huang, Nian-Sheng Tzeng, Liang-Jen Wang, I Hui Lee, Tzu-Yun Wang, Kao Chin Chen, Yen Kuang Yang, Jau-Shyong Hong, Ru-Band Lu

**Affiliations:** Department of Psychiatry, Kaohsiung Veterans General Hospital, Kaohsiung, Taiwan (Dr S-Y Lee); Department of Psychiatry, National Cheng Kung University, Tainan, Taiwan (Drs S-Y Lee, P Chen, I Lee, T-Y Wang, K Chen, Yang, and Lu); Department of Neurology, School of medicine, Kaohsiung Medical University, Kaohsiung, Taiwan (Dr S-L Chen); Institute of Allied Health Sciences, National Cheng Kung University, Tainan, Taiwan (Drs Chang and Lu); Laboratory of Neurobiology, NIH/NIEHS, Research Triangle Park, NC (Drs Chu and S-H Chen); Department of Psychiatry, Tri-Service General Hospital, National Defense Medical Center, Taipei, Taiwan (Drs Huang and Tzeng); Department of Child and Adolescent Psychiatry, Kaohsiung Chang Gung Memorial Hospital and Chang Gung University College of Medicine, Kaohsiung, Taiwan (Drs L-J Wang, T-Y Wang, and Hong); Institute of Behavioral Medicine, College of Medicine and Hospital, National Cheng Kung University, Tainan, Taiwan (Dr Lu); Addiction Research Center, National Cheng Kung University, Tainan, Taiwan (Dr Lu); Center for Neuropsychiatric Research, National Health Research Institutes, Miaoli, Taiwan (Dr Lu).

**Keywords:** cytokine, dextromethorphan, methadone maintenance therapy, opioid dependence, Dextromethorphan, Heroin, Cytokines

## Abstract

**Background:**

Low-dose dextromethorphan (DM) might have anti-inflammatory and neurotrophic effects mechanistically remote from an NMDA receptor. In a randomized, double-blind, controlled 12 week study, we investigated whether add-on dextromethorphan reduced cytokine levels and benefitted opioid-dependent patients undergoing methadone maintenance therapy (MMT).

**Methods:**

Patients were randomly assigned to a group: DM60 (60mg/day dextromethorphan; n = 65), DM120 (120mg/day dextromethorphan; n = 65), or placebo (n = 66). Primary outcomes were the methadone dose required, plasma morphine level, and retention in treatment. Plasma tumor necrosis factor (TNF)-α, C-reactive protein, interleukin (IL)-6, IL-8, transforming growth factor–β1, and brain-derived neurotrophic factor (BDNF) levels were examined during weeks 0, 1, 4, 8, and 12. Multiple linear regressions with generalized estimating equation methods were used to examine the therapeutic effect.

**Results:**

After 12 weeks, the DM60 group had significantly longer treatment retention and lower plasma morphine levels than did the placebo group. Plasma TNF-α was significantly decreased in the DM60 group compared to the placebo group. However, changes in plasma cytokine levels, BDNF levels, and the methadone dose required in the three groups were not significantly different.

**Conclusions:**

We provide evidence—decreased concomitant heroin use—of low-dose add-on DM’s efficacy for treating opioid-dependent patients undergoing MMT.

## Introduction

Opioid dependence is usually characterized by repetitive drug-seeking and drug-taking behaviors. Due to the high relapse rate, opioid dependence causes severe public health consequences. For opioid detoxification, a well-established first-line strategy by the American Psychiatric Association is to replace the opioid with methadone and then to gradually taper the patient off methadone (Kleber et al., 2007). Current treatment for opioid dependence in practice, including agonist maintenance, which uses methadone or buprenorphine to taper users off opioids, provides the most effective long-term approach because it contributes to long-lasting stabilization of the opioid dependency (Kleber, 2007). Methadone maintenance therapy (MMT), which substitutes methadone, a synthetic opioid, for abused opioids and blocks their effects, has been suggested by the World Federation of Societies of Biological Psychiatry Guidelines as effective treatment for opioid dependence (Soyka et al., 2011). Relapses of opioid use and abuse often occur after MMT is discontinued. Although MMT that includes an adjunctive therapy might be more beneficial than MMT that uses methadone alone, the current crop of combined therapies appears not to be comprehensive enough for treating opioid dependence. Furthermore, methadone tolerance, a need for increasing doses of methadone to achieve a treatment response, is frequent in long-term treatment (Trafton et al., 2006). Treating opioid-dependent patients remains a challenge and the methods need to be improved. Therefore, developing effective adjuvant therapeutic interventions for opioid-dependent patients during long-term MMT should be encouraged.

Opioids cause oxidative stress and inflammatory responses. *In vivo* and *in vitro* human and animal studies show that opioid abuse has adverse immunomodulatory effects on innate and adaptive immune responses (Thomas et al., 1995). *In vitro* studies (Peterson et al., 1994; Kapasi et al., 2000; Zubelewicz et al., 2000) report that acute morphine treatment influences the production of tumor necrosis factor (TNF)-α and interleukin (IL)-6. Subcutaneous and intra-cerebroventricular morphine also elevate serum IL-6 levels in rats (Houghtling and Bayer, 2002). Increased cytokine expression levels have been detected in noradrenergic locus coeruleus cells in the brains of opioid-dependent patients (Dyuizen and Lamash, 2009). However, many confounding factors (e.g. chronic infections, hepatitis, HIV, and intravenous injections with contaminated substances) also affect the immune system of opioid-dependent patients. Therefore, the direct link between opioid dependence and the immune system remains controversial. In contrast, decreased serum concentrations of brain-derived neurotrophic factor (BDNF) and nerve growth factor have been detected in chronic heroin users (Angelucci et al., 2007). Another study (Graham et al., 2007) showed that the increase of BDNF in the nucleus accumbens was closely related to dependence on cocaine and other drugs, and to a dependency relapse. Furthermore, BDNF seems to be involved in long-term behavioral adaptation caused by drug dependence (Bolanos and Nestler, 2004). These findings suggest that chronic opioid use stimulates cytokine production in both the peripheral and central nervous systems, which causes systemic inflammation, neuroinflammation, and subsequent neuron damage (Bruce-Keller et al., 2008). Treatment combining anti-inflammatory and neuroprotective agents might be more beneficial than current management strategies without these agents.

Dextromethorphan (DM) is an antitussive drug used for more than 50 years (Turchan-Cholewo et al., 2009). Studies suggest that DM may be useful for treating opioid dependence (Bisaga and Popik, 2000) by reducing the development of opiate tolerance (Wu et al., 2009), reducing withdrawal symptoms during acute detoxification from opioids (Lin et al., 2014), and inhibiting conditioned reactions to drug-related cues (O’Brien et al., 1998). As an N-methyl-D-aspartate (NMDA) receptor antagonist, it is useful at a high dose (480mg/day) for reducing methadone tolerance in opioid-dependent patients (Cornish et al., 2002). DM also protects monoaminergic neurons against inflammation-mediated degeneration (Liu and Hong, 2003) and endotoxicity (Liu and Hong, 2003; Zhang et al., 2004, 2005). The evidence suggests that DM offers promising neuroprotective and anti-inflammatory benefits in neurodegenerative disorders. Because of these findings that DM benefits the autoimmune system, we hypothesized that adding DM to MMT would therapeutically benefit opioid-dependent patients because of its reported anti-inflammatory and neuroprotective effects.

We conducted a double-blind, placebo-controlled study to evaluate the efficacy of add-on low-dose DM (60 and 120mg/day) in opioid-dependent patients undergoing MMT. We hypothesized that DM would reduce methadone tolerance and therefore both reduce the dose needed, the craving for heroin, and inflammation and increase opioid withdrawal relief in opioid-dependent patients.

## Methods

### Patient Selection

The research protocol was approved by the Institutional Review Board for the Protection of Human Subjects at National Cheng Kung University Hospital (NCKUH). After the study had been completely described to the participants, they all signed written informed consent forms.

Opioid-dependent patients were recruited from the NCKUH MMT program. Each was initially evaluated in an interview by an attending psychiatrist. Those who were diagnosed with opioid dependence and did not meet the exclusion criteria were given an explanation of the study protocol. Those who agreed to participate in the study and signed informed consents subsequently underwent a more detailed structural interview by a research team member trained in using the Diagnostic and Statistical Manual of Mental Disorders, fourth edition (DSM-IV) criteria and the Chinese Version of the Mini International Neuropsychiatric Interview (MINI; Sheehan et al., 1998). The MINI is a short (20–30 minutes) structured diagnostic interview that evaluates Axis I and II diagnoses with good validity and reliability (Sheehan et al., 1998). We chose the MINI because it is difficult for opioid-dependent patients to complete a 4 to 6 hour structured interview, such as the Chinese Version of the Modified Schedule of Affective Disorder and Schizophrenia–Lifetime (Endicott and Spitzer, 1978). Inclusion criteria were men and women 18–65 years old who met the DSM-IV criteria for current opioid dependence and used opioids daily. Exclusion criteria were antisocial personality disorder, a cognitive disorder, or a major or minor mental illness other than opioid dependency. Other exclusion criteria were being pregnant or nursing an infant, having taken any anti-inflammatory medications within 1 week before the study, or having a history of one or more uncontrolled chronic major physical conditions such as diabetes mellitus or hypertension.

### Study Design

Participants were randomly assigned to one of three treatment groups: placebo (methadone + one daily placebo capsule), DM60 (methadone + one daily 60mg sustained-release dextromethorphan capsule), or DM120 (methadone + one daily 120mg sustained-release dextromethorphan capsule) for 12 weeks. Methadone doses were increased or decreased by 5mg when necessary in response to each participant’s clinical situation. The primary outcome of the study was to compare the methadone dose required, retention rates, and concomitant opioid use (plasma morphine) of participants in the 12-week trial. The methadone doses required and plasma morphine levels were recorded at baseline and on day 7 of weeks 1, 4, 8, and 12.

Ten milliliters of whole blood was withdrawn from the antecubital vein of each patient at baseline and on day 7 of weeks 1, 4, 8, and 12. Plasma, which was isolated from the whole blood after it had been centrifuged at 3000g for 15min at 4°C, was immediately stored at −80°C. The levels of the immunological parameters (TNF-α, C-reactive protein [CRP], IL-6, IL-8, and TGF-β1) were quantified using an antibody pair assay system (Flexia; BioSource Intl.). A BDNF kit (Quantikine Human BDNF kit; R&D Systems) and an enzyme-linked immunosorbent assay reader (SpectraMax-M2; Molecular Devices) were used to analyze the plasma BDNF level. Samples were processed and data analyzed according to the manufacturer’s instructions. Plasma morphine and dextromethorphan concentrations were determined using high-performance liquid chromatography (HPLC) as previously described (Chen et al., 2005). The plasma was filtered (Amicon Microcon YM-3 centrifugal filters; Millipore; 3000 molecular weight [MW] cutoff) at 17 800 × g for 40min at 4°C. The recovery rate of the filtration was 100%. The filtered sample was then injected into the HPLC system to measure free-form morphine and dextromethorphan concentrations.

### Statistical Analysis

The demographic and clinical characteristics of the patients and their baseline methadone dose, cytokine levels, and BDNF levels were compared between groups using one-way analyses of variance (ANOVA) for continuous variables and χ^2^ tests for categorical variables. Data are means ± standard deviation. Arithmetic transformations were used to produce approximately normal distributions for further analysis; log (*x* + 1) was used for cytokine levels. Potential prognostic factors included the treatment duration (0–12 weeks), dextromethorphan dose, gender, and age. Because there were repeated assessments, multiple linear regression—controlled for time effects, age, and gender, and with the generalized estimating equationmethod (Zeger et al., 1988)—was used on longitudinal outcomes (methadone doses, plasma morphine level, cytokine levels, and BDNF levels) to evaluate the possible effects of the prognostic factors on the response values. A repeated-measures ANOVA was used to test the trends of plasma morphine levels in the three opioid-dependent groups. The retention rate was estimated using the Kaplan-Meier product-limit estimate method, and survival curves for the two groups were compared using the Wilcoxon rank sum test. SPSS 18.0 for Windows was used for statistical computations. Significance was set at p < 0.05.

## Results

**Figure 1.**
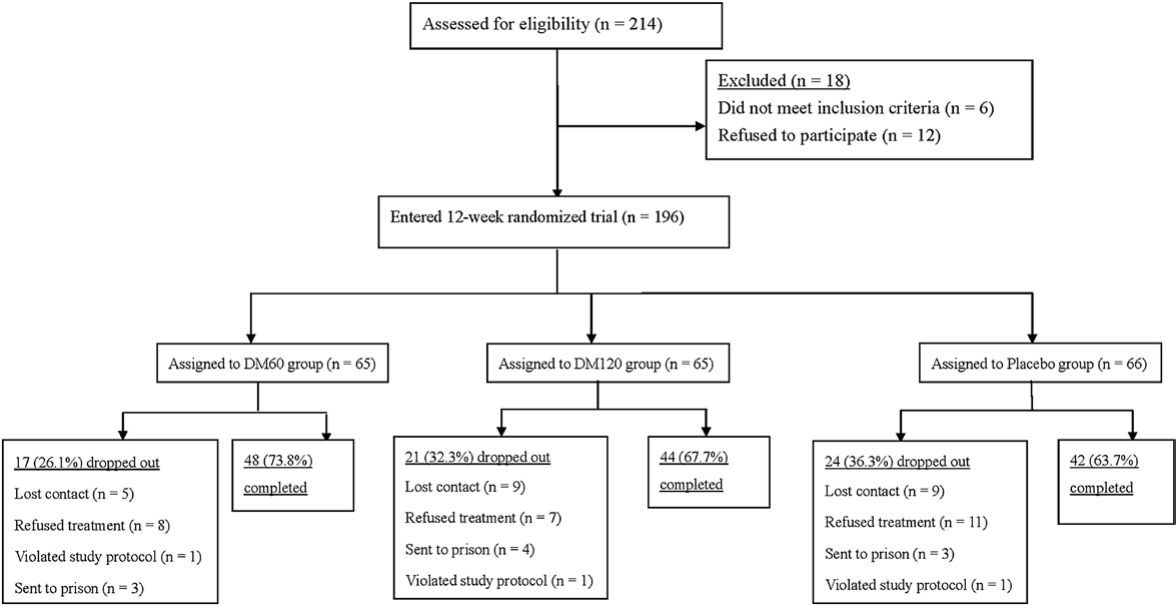
CONSORT diagram showing the disposition of patients in the study. DM, dextromethorphan.

A total of 214 opioid-dependent patients were screened for eligibility (see Figure 1 for the CONSORT Flow Diagram). Out of those, 18 declined to participate (did not complete the evaluation, did not meet recruiting criteria, or were not interested in treatment other than methadone). The remaining 196 opioid-dependent participants entered the study and were randomly assigned to the DM60 group (n = 65), DM120 group (n = 65), or the placebo group (n = 66) for 12 weeks. At completion of the double-blind phase, 134 (68.4%) participants remained and 62 (31.6%) had dropped out (DM60: n = 17; DM120: n = 21; placebo: n = 24). Their reasons for discontinuing the study were: loss of follow-up for an unknown reason (DM60: n = 5; DM120: n = 9; placebo: n = 9), refused treatment (DM60: n = 8; DM120: n = 7; placebo: n = 11), violation of protocol (DM60: n = 1; DM120: n = 1; placebo: n = 1), and remanded to prison during treatment (DM60: n = 3; DM120: n = 4; placebo: n = 3). Two adverse events were reported in the placebo group (nausea, chest tightness).

**Figure 2.**
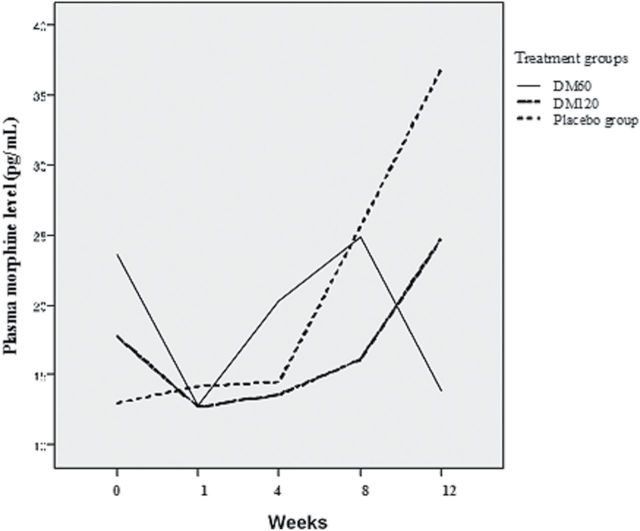
Change in the mean plasma morphine levels in the DM60, DM120, and placebo groups after 12 weeks of treatment. DM, dextromethorphan.

The demographic and clinical characteristics, baseline methadone dose, and BDNF and cytokine levels of the patients were similar in all patient groups at baseline, but all cytokine levels were distributed erratically and showed a significant level of positive skew (Table 1). We used multiple linear regressions to analyze the treatment effects. The changes in the required methadone dose after 12 weeks of treatment were not significantly different between groups. Plasma morphine was significantly lower in the DM60 group (*p* = 0.003), but not the DM120 group, compared with the placebo group (Figure 2; Table 2). The plasma morphine level in the DM120 group did not change significantly (F = 0.780, *p* = 0.539), but the placebo group showed a significant trend of increasing plasma morphine levels (F = 3.387, *p* = 0.010), and the DM60 group showed a significant trend of decreasing plasma morphine levels (F = 3.051, *p* = 0.018).

**Table 1.** Baseline and Endpoint Characteristics of Opioid-Dependent Patients Taking Dextromethorphan or a Placebo

	Baseline					Endpoint			
Characteristics	DM60	DM120	Placebo	*p*		DM60	DM120	Placebo	*p*
Number (n)	65	65	66			48	44	42	
Gender (M/F)	54/11	54/11	62/4	0.114		42/6	37/7	40/2	0.243
Age (years) [mean ± SD]	39.88±6.86	38.91±6.97	38.67±7.38	0.586		40.74±6.80	39.20±7.44	39.67±8.06	0.597
TNF-α (pg/mL) [mean ± SD]	4.48±3.41	3.85±3.94	3.76±4.49	0.654		3.24±2.94	2.97±2.63	3.16±4.18	0.919
CRP (ng/mL) [mean ± SD]	1878.74±2182	2643.84±3331	2428.49±2558	0.403		1745.66±1657	2264.69±2651	2513.39±2671	0.161
IL-6 (pg/mL) [mean ± SD]	2.28±1.57	2.43±2.20	2.40±2.10	0.939		1.96±1.70	1.78±1.26	2.06±1.48	0.682
IL-8 (pg/mL) [mean ± SD]	7.98±12.81	5.87±4.92	5.51±6.55	0.343		4.63±4.70	4.17±4.00	2.90±2.25	0.101
TGF-β1 (ng/mL) [mean ± SD]	31.92±15.49	38.94±22.00	31.39±17.54	0.099		26.53±14.29	27.40±12.67	24.99±15.17	0.730
BDNF (ng/mL) [mean ± SD]	11.85±8.25	13.15±6.65	10.58±5.43	0.185		8.50±4.43	10.10±5.20	8.35±5.19	0.194
Methadone dose (mg) [mean ± SD]	41.4±25.1	42.9±22.7	44.2±28.4	0.810		42.8±21.3	43.8±23.3	48.2±28.6	0.534
Plasma opioid level (pg/mL)	23.61±57.26	17.74±40.06	12.99±32.87	0.535		13.86±20.57	24.72±52.09	36.84±64.40	0.108

BDNF, brain-derived neurotrophic factor; CRP, C-reactive protein; DM60, dextromethorphan 60mg/day; DM120, dextromethorphan 120mg/day; IL-6, interleukin 6; IL-8, interleukin 8; SD, standard deviation; TNF-α, tumor necrosis factor-α; TGF-β1, transforming growth factor -β1.

**Table 2.** Changes in Methadone Dose and Cytokine Levels From Baseline After 12 Weeks of Dextromethorphan or Placebo Treatment in Opioid-Dependent Patients

	DM60			DM120		
Parameter	B	Wald χ^2^	*p*	B	Wald χ^2^	*p*
Methadone dose required^1^	2.129	2.132	0.144	2.485	2.956	0.086
Plasma morphine level^2^	−0.104	8.723	0.003**	−0.051	1.658	0.198
TNF-α (pg/mL)^3^	−0.681	3.881	0.049*	−0.282	1.661	0.197
CRP (pg/mL)^3^	−0.052	2.053	0.152	−0.019	0.269	0.604
IL-6 (pg/mL)^3^	0.016	0.350	0.554	0.007	0.059	0.808
IL-8 (pg/mL)^3^	0.013	0.079	0.778	−0.041	1.037	0.309
TGF-β1 (pg/mL)^3^	0.005	0.008	0.929	0.001	< 0.001	0.984
BDNF^3^	0.023	0.648	0.421	0.018	0.499	0.480

BDNF, brain-derived neurotrophic factor; CRP, C-reactive protein; DM60, dextromethorphan 60mg/day; DM120, dextromethorphan 120mg/day; IL-6, interleukin 6; IL-8, interleukin 8; TNF-α, tumor necrosis factor-α; TGF-β1, transforming growth factor β1.

^1^Controlled for treatment course, plasma opioid level, gender, and age; ^2^Controlled for treatment course, methadone dose, gender, and age; ^3^Controlled for treatment course, plasma opioid level, methadone dose, gender, and age. Reference group is placebo group. *p < 0.05, **p < 0.01.

**Figure 3.**
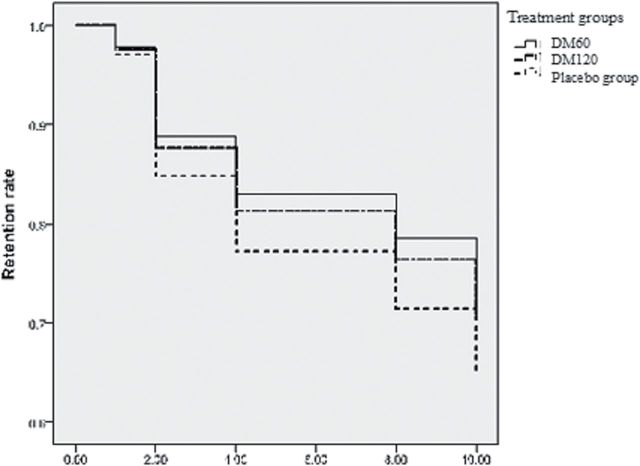
The retention rates in the DM60, DM120, and placebo groups after 12 weeks of treatment. DM, dextromethorphan.

The DM60 group also had a significantly (*p* = 0.049) lower TNF-α level than did the placebo group, but other cytokine levels and BDNF levels were not significantly different. However, if we correct for multiple comparisons, setting *p* < 0.025 as significant, the decrease of TNF-α level in the DM60 group became non-significant when compared with the placebo group. In contrast, there was no significant difference between the DM120 and placebo groups. The retention rate was significantly higher in the DM60 group, but not the DM120 group, than in the placebo group (Table 3; Figure 3).

**Table 3.** Hazard Ratio of Dropout During the Trial

	Dropout rate	Adjusted hazard ratio model	
Treatment Group	n/Total n (%)	Exp (B)	*p*
DM60	17/65 (26.1)	0.718	0.013
DM120	21/65 (32.2)	0.801	0.083
Placebo	24/66 (36.4)	1 (Ref)	

DM60, dextromethorphan 60mg/day; DM120, dextromethorphan 120mg/day; Exp (B), odds ratio; (Ref), reference group. Cox proportional hazards model; n = 62.

## Discussion

We found that add-on dextromethorphan (60mg/day; DM60) was significantly more effective than placebo for attenuating plasma morphine levels. The retention rate was significantly higher in the DM60 group than in the placebo group. Our findings support the notion that low-dose dextromethorphan is well tolerated by opioid-dependent patients and can reduce concomitant opioid use. We found that DM60 was significantly more effective than was placebo for reducing TNF-α levels, but not for modulating BDNF levels or levels of the inflammatory cytokines CRP, IL-6, IL-8, and TGF-β in opioid-dependent patients. There were no significant differences in the levels of plasma opioids, plasma cytokines, or plasma BDNF, nor was there a significant difference in treatment retention between the DM120 and placebo groups.

One of our primary outcomes was retention in treatment, because dropping out of treatment is likely to be associated with a relapse to opioid use. Furthermore, concomitant opioid use is common even for opioid-dependent patients undergoing MMT. Therefore, the plasma morphine level was another primary outcome. After 12 weeks of treatment with MMT plus add-on low-dose dextromethorphan (DM60 or DM120), we found that the retention rate was significantly higher in the DM60 group. In addition, the plasma opioid level was significantly lower in the DM60 group than in the placebo group. There were, however, no differences between the three groups in the doses of methadone required. From these findings, we conclude that 60mg per day of add-on dextromethorphan effectively attenuates concomitant opioid use and reduces the dropout rate in opioid-dependent patients, but does not increase tolerance to or the requirement for methadone compared with placebo. Our findings support the notion that a reduction in the plasma morphine level and no increase in the required dose of methadone indicates that the patient’s opioid withdrawal symptoms were less severe in the DM60 group than in the placebo group. We hypothesize that add-on DM60 decreases both psychological (craving) and physiological (withdrawal symptoms) dependence for opioid-dependent patients undergoing MMT.

We also found that add-on DM60 was beneficial for attenuating plasma TNF-α levels in opioid-dependent patients. However, a longer follow-up period (at least 6 months) is necessary in future experiments to confirm our finding. When used at higher doses (375–480mg/day), dextromethorphan attenuated the symptoms of opioid withdrawal (Bisaga et al., 1997) and reduced tolerance to methadone (Cornish et al., 2002). As an adjunct to clonidine (240mg/day) in a recent clinical study (Lin et al., 2014), dextromethorphan seemed to mitigate the symptoms of opioid withdrawal. However, most studies on dextromethorphan’s therapeutic effects on opioid dependence have small study populations that have yielded controversial findings. Other researchers have attributed dextromethorphan’s beneficial effects against substance abuse to its NMDA blocker effect (Thomas et al., 1995; Dyuizen and Lamash, 2009). Although dextromethorphan is known as a weak NMDA receptor antagonist (ED_50_: 5–50 μM; Church et al., 1994), in the current study the low plasma dextromethorphan concentrations (20.2±47.5ng/mL: data not shown) in the DM60 group were insufficient to block NMDA receptors. We previously showed (Li et al., 2005) that femtomolar concentrations of dextromethorphan protected mesencephalic dopaminergic neurons in a neuron-glia culture. Other studies (Liu et al., 2003; Zhang et al., 2004, 2005, 2006; Li et al., 2005) have also reported that dextromethorphan protected dopaminergic neurons against 1-methyl-4-phenyl-1,2,3,6-tetrahydropyridine (MPTP) and lipopolysaccharides-induced dopaminergic neuron damage *in vitro* and *in vivo*. We hypothesize that the decline in TNF-α levels in the current study were the result of dextromethorphan’s anti-inflammatory effect, not its function as an NMDA-receptor blocker. Additional mechanistic studies are necessary to confirm this hypothesis.

Dextromethorphan is metabolized primarily by the cytochrome P450 (CYP) 2D6 isozyme, but methadone inhibits CYP2D6 (Lotrich et al., 2005). There is, therefore, a potential interaction between dextromethorphan and methadone. Combining dextromethorphan and methadone treatment might increase the methadone concentration and decrease the methadone dose required. Although there were no differences in the required methadone dose after 12 weeks of treatment between the DM60, DM120, and placebo groups, a significant reduction in the plasma morphine level was found in the DM60 group, indicating that the patients’ opioid withdrawal symptoms were less severe in the DM60 group than the placebo group. We propose that the reduction of opioid withdrawal symptoms may be related to DM’s effect in increasing plasma methadone concentration. Further research evaluating the effect of DM on plasma methadone levels is warranted. Because genetic variants of the CYP2D6 isozyme cause varying levels of metabolic activity, the association between the CYP2D6 polymorphism and the metabolizing activities of combined dextromethorphan and methadone treatment should be evaluated in additional studies.

In the present study, there was significant improvement only in the low-dose DM60 group compared with the placebo group. One possible explanation for our findings is that dextromethorphan has a narrow therapeutic window in Parkinson’s disease (60–120mg/day; Verhagen Metman et al., 1998). Moreover, an inverted U-shaped dose-response curve of medication for dopamine-related disorders has been reported (Papa and Chase, 1996; Cools and D’Esposito, 2011). Hence, low-dose dextromethorphan may be more effective than high-dose. However, a more detailed dose-response curve of add-on for treating opioid dependence warrants further study. A second explanation could be that the polymorphism of the CYP2D6 enzyme related to dextromethorphan metabolism may also influence the plasma concentration of dextromethorphan and therefore confound the search for an effective dose of dextromethorphan. A more careful investigation controlling for dextromethorphan metabolism while searching for the most effective dose is needed.

When treating neuropsychiatric disorders, being able to identify and quantify peripheral biomarkers for a diagnosis or for monitoring treatment responses remains a clinical goal. Some studies (Angelucci et al., 2007; Ghazavi et al., 2013a, 2013b) have suggested that changes in proinflammatory cytokines and BDNF may be related to the pathophysiology of opioid dependence. In the present study, we found that add-on dextromethorphan was no more effective than was placebo for modulating IL-6, IL-8, IL-1β, CRP, and BDNF levels in opioid-dependent patients. Furthermore, dextromethorphan was no more effective than was placebo for attenuating the required methadone dose, which indicates the patient’s tolerance of methadone. We suspect that 12 weeks may not be long enough to detect other clinical and immunological improvements. At least 6 months of treatment may be needed.

Our study has some limitations. First, it was undoubtedly too short and our study populations too small to confirm our positive findings. Furthermore, if we correct for multiple comparisons, our positive findings for dextromethorphan’s beneficial effects on attenuating TNF-α levels may not hold up. In addition, we did not explore other factors, such as smoking and weight, which might influence the effects of dextromethorphan. Nor did we test for other plasma opioids as an outcome measure. In addition, there was no objective base for the decision to increase methadone dose. Second, we measured plasma cytokines because other studies suggested that changes in peripheral cytokine secretion might indicate changes in central levels. However, like other studies (e.g. Pan and Kastin, 1999), we were unable to arrive at a definitive conclusion about this. Moreover, Lessenger and Feinberg (2008) suggested that dextromethorphan is likely to be abused because it produces a dissociative effect similar to that of phencyclidine and ketamine at higher doses. Therefore, although the present study suggests that add-on low-dose dextromethorphan has therapeutic benefits, follow-up studies on the abuse of this drug by opioid-dependent patients may be needed. Third, many confounding factors (e.g. chronic infections, hepatitis, HIV, and intravenous injections with contaminated substances) also affect the immune system of opioid-dependent patients. Because almost all the opioid-dependent patients in the methadone clinic have hepatitis C, we did not exclude them or patients with HIV. Given the inclusion of patients with chronic inflammatory disorders (hepatitis C, HIV infections), the immunological data of the current research should be interpreted with caution. Finally, because the present study was a fixed-dose comparison without dose-assessment trials, the definitive effects of add-on dextromethorphan and their clinical efficacy require additional studies.

In conclusion, DM60 decreased plasma opioid levels and prolonged the treatment retention rate of opioid-dependent patients undergoing MMT without increasing the methadone dose required. It also significantly reduced plasma TNF-α levels but had little effect on other cytokines. Our data support the efficacy of low-dose dextromethorphan in treating opioid-dependent patients on MMT. Low-dose dextromethorphan might be a feasible adjuvant therapy for decreasing concomitant opioid use, mitigating opioid withdrawal symptoms, and attenuating inflammation.

## Statement of Interest

All authors declare that they have no conflicts of interest.
